# Microbe-metabolite interaction networks, antibiotic resistance, and in vitro reconstitution of the penile prosthesis biofilm support a paradigm shift from infection to colonization

**DOI:** 10.1038/s41598-023-38750-1

**Published:** 2023-07-17

**Authors:** Glenn T. Werneburg, Scott D. Lundy, Daniel Hettel, Petar Bajic, Bradley C. Gill, Ava Adler, Sromona D. Mukherjee, Hadley M. Wood, Kenneth W. Angermeier, Daniel A. Shoskes, Aaron W. Miller

**Affiliations:** Department of Urology, Cleveland Clinic Foundation, Glickman Urological and Kidney Institute, Cleveland, OH 44195 USA

**Keywords:** Clinical microbiology, Sexual dysfunction, Biofilms

## Abstract

To understand differences between asymptomatic colonized and infected states of indwelling medical devices, we sought to determine penile prosthesis biofilm composition, microbe-metabolite interaction networks, and association with clinical factors. Patients scheduled for penile prosthesis removal/revision were included. Samples from swabbed devices and controls underwent next-generation sequencing, metabolomics, and culture-based assessments. Biofilm formation from device isolates was reconstituted in a continuous-flow stir tank bioreactor. 93% of 27 analyzed devices harbored demonstrable biofilm. Seven genera including *Faecalibaculum* and *Jeotgalicoccus* were more abundant in infected than uninfected device biofilms (*p* < 0.001). Smokers and those with diabetes mellitus or cardiac disease had lower total normalized microbial counts than those without the conditions (*p* < 0.001). We identified microbe-metabolite interaction networks enriched in devices explanted for infection and pain. Biofilm formation was recapitulated on medical device materials including silicone, PTFE, polyurethane, and titanium in vitro to facilitate further mechanistic studies. Nearly all penile prosthesis devices harbor biofilms. *Staphylococcus* and *Escherichia*, the most common causative organisms of prosthesis infection, had similar abundance irrespective of infection status. A series of other uncommon genera and metabolites were differentially abundant, suggesting a complex microbe-metabolite pattern–rather than a single organism–is responsible for the transition from asymptomatic to infected or painful states.

## Introduction

Penile prostheses are an effective treatment for erectile dysfunction with excellent long-term satisfaction for patients and partners. The penile prosthesis, which generally consists of two inflatable cylinders and a pump connected to a reservoir, is constructed of various materials including silicone, polyurethane, polytetrafluoroethylene (PTFE), and titanium. Infection remains among the most feared complications associated with penile prosthesis placement and is associated with significant morbidity and cost exceeding six times the cost of initial uncomplicated prosthesis placement^1^. Risk-modifying factors to reduce infection have been implemented, including antibiotic-coatings or device impregnation, both of which have significantly reduced infection risk^1,2^. These generally include silicone impregnation with slow-release rifampin and minocycline or a hydrophilic surface that binds antibiotic from solutions chosen by the surgeon^3^. Despite these advances, 1–3% risk of device infection remains^1,3–5^.

Biofilms are complex communities of bacteria adherent to one another and an underlying surface. They consist of microbes and their breakdown products (metabolites), and extracellular matrix components. Next-generation sequencing (NGS) allows for the high-throughput analysis of microbial composition. It may be particularly useful in the context of implanted medical devices, which are generally associated with low microbial biomass even in the presence of infection. NGS has been studied for use in orthopedic periprosthetic joint infections, and has favorable diagnostic accuracy in patients in whom device cultures are negative and in whom there is history of antibiotic use^6,7^. NGS has also been studied for the analysis of biofilm composition in device types across medical specialties including plastic surgery^8^, cardiac surgery^9^, critical care medicine^10^. Within urology, NGS technology has been used to characterize the composition of biofilm on devices that dwell in different anatomical niches including ureteral stents^11^ and artificial urinary sphincters^12^. While microbial profiles may differ in the context of device-associated infection^13^, how specific microbe-metabolite communities relate to infection and patient factors remains poorly understood. Further, whether microbes isolated from prostheses have a propensity to reconstitute biofilm in vitro–and to what degree this relies on specific materials–has yet to be determined.

We hypothesized that penile prosthesis devices would harbor unique microbial biofilms that vary according to infection status and patient factors. To test this, we determined the composition and natural history of biofilm formation on penile prosthesis devices. Next, we characterized microbe-metabolite interaction networks to determine associations between these patterns and clinical factors, including infection. Finally, we reconstituted biofilm from bacterial strains isolated from devices for biofilm formation in vitro on a series of device materials. The goal of this work is to develop a new understanding of the transition from colonized to infected states and open new avenues for the rational design of future anti-infective materials and coatings for implantable devices.

## Subjects and methods

### Study sample

Patients scheduled for penile prosthesis removal or revision were identified and consented via IRB-approved protocol (20–415). Individuals 18 years of age or older were included. Those who were unable to provide informed consent were excluded. Prior to surgically accessing the device, a swab of subcutaneous tissue was obtained as a negative control. The first-accessed-portion of the device was swabbed with three standard culture swabs to sample the device biofilm (1: 16 s next-generation sequencing; 2: metabolomics; 3: standard culture), with care taken to avoid contamination from the remainder of the operative field. Samples were collected under direct supervision by a study investigator, and demographics and patient factors were captured in a prospectively-accrued database.

Sample processing, sequencing, detection of antibiotic resistance and biofilm-associated genes, metabolite analysis (metabolomics), bioinformatics, biofilm assays, scanning electron microscopy, and statistical analysis methodology are included in Supplementary Material. The study was conducted in accordance with the Declaration of Helsinki. All experimental protocols were approved by the Cleveland Clinic Institutional Review Board (IRB). Informed consent was obtained from all subjects. Devices were classified as “explanted for infection” if there were local and/or constitutional signs or symptoms suggestive of implant-associated infection. These included site erythema, fluctuance, purulent drainage, fever, or rigors. Devices expanted for pain were classified as such if there was pain that prompted device removal, but no other signs or symptoms suggestive of infection. Microbial growth on clinical culture of devices or surrounding tissue was not a criterion used for infection.

### Ethics of approval and patient consent

Cleveland Clinic IRB 20–415.

## Results

A total of 27 penile prosthesis devices explanted from individuals for any cause were included and analyzed in the study. Mean age at device explant was 64 (standard deviation 11.5) years, and mean device indwelling time was 4.8 (5.0) years (Table 1). Eleven (41%) of the 27 patients from whom devices were explanted had a diagnosis of diabetes mellitus, and 11% were current smokers. Four patients (14.8%) had device infection as the indication for explant, and four patients had device-associated pain requiring explant (14.8%). Eighteen patients (66.7%) had the device removed for malfunction, and one patient (3.7%) had the device removed due to lack of satisfaction with the device. Eighteen (66.7%) of devices were minocycline-rifampin impregnated, 4 (14.8%) were those dipped in an antibiotic solution determined by the surgeon, and 5 (18.5) of the devices were malleable penile prostheses. Of the four infected devices, three were minocycline-rifampin impregnated and one was a malleable device. There was no statistical difference in infection rate across device types in this cohort (*p* = 1.0, Fisher’s exact test).

**Table 1:** Demographics from individuals from whom penile prostheses were included.

Factor	Mean (SD) or n (%) (n = 27)
Age (years)	64 (11.5)
Race	
White	21 (77.8)
Black	6 (22.2)
Body mass index (kg/m^2^)	29.4 (5.2)
Diabetes mellitus	11 (40.7)
Cardiac disease	10 (37.0)
Current smoker	3 (11.1)
Penile prosthesis type	
AMS	18 (66.7)
Coloplast	4 (14.8)
Malleable	5 (18.5)
Device indwelling time (years)	4.8 (5.0)
Operative time at placement (minutes)	124.2 (32.9)
Indication for device removal	
Device-associated infection	4 (14.8)
Device-associated pain	4 (14.8)
Device malfunction	18 (66.7)
Unsatisfied with device	1 (3.7)
Antibiotics during 30 days prior to device removal	7 (26.9)^a^

^a^Antibiotic information for one individual was unknown.

### Microbial composition

**Figure 1:**
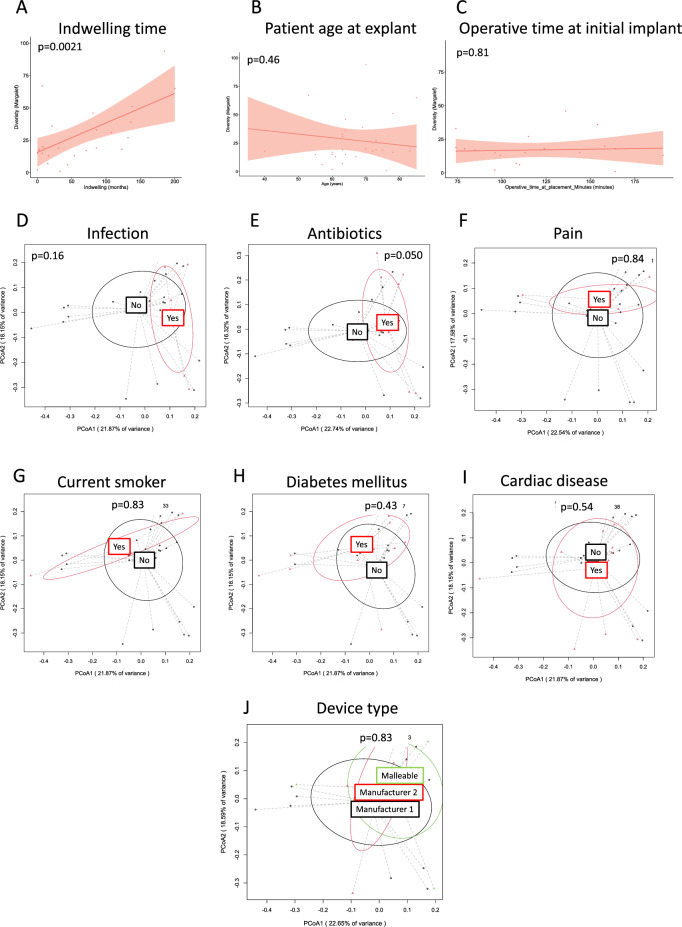
Microbial diversity differs by indwelling time and antibiotic use in the past 30 days. Alpha (**A**–**C**) or Beta (**D**–**J**) diversity is indicated. Panels are labeled with respective patient factors. *p*-values are indicated in each respective panel. Ellipses indicated standard deviation.

Rarefaction analysis found 25 (93%) of 27 penile prosthesis samples adequately captured microbiota diversity. The alpha-diversity of biofilms increased with indwelling time (*p* = 0.002) (Fig. 1A). Alpha-diversity did not differ by age or operative time at device placement (Fig. 1B,C). Beta-diversity was similar based on device-associated infection status (Fig. 1D, *p* = 0.16). Beta-diversity differed between those who had antibiotic prescription within 30 days of surgery versus those who did not (Fig. 1E, *p* = 0.050), but did not differ based on device-associated pain (Fig. 1F, *p* = 0.84). Diagnoses of diabetes mellitus, cardiac disease, or current smoking status, and device type were not associated with significant differences in microbial diversity (Fig. 1G–J).

**Figure 2:**
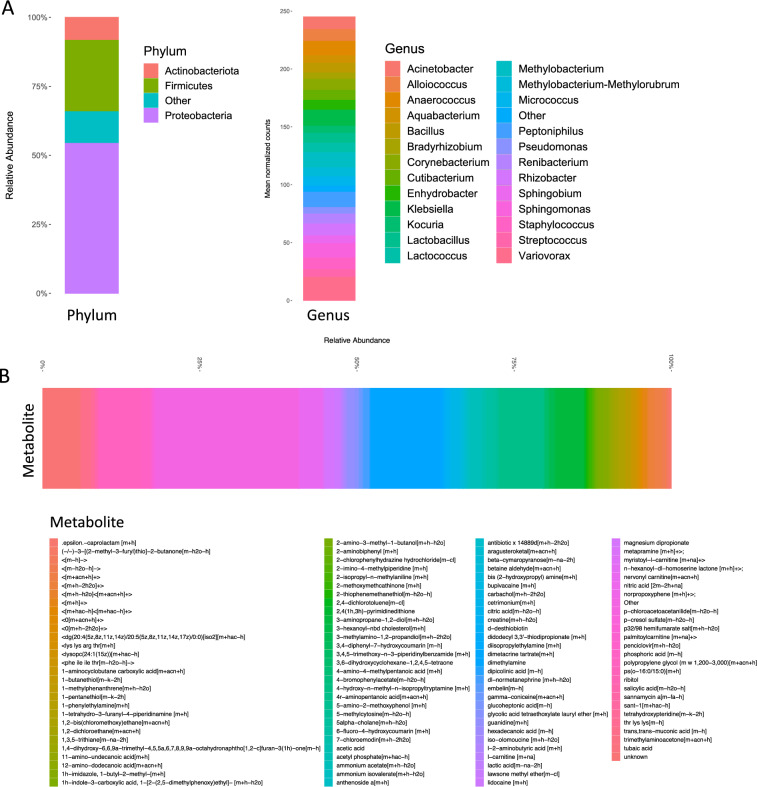
Microbe and metabolite composition on penile prosthesis biofilms. (**A**) Relative abundance of microbial phyla (left) and normalized counts of microbial taxa (right) on device biofilms as detected by 16 s sequencing. (**B**) Metabolite relative abundance as detected using liquid chromatography/tandem mass spectroscopy (LC–MS-MS).

The predominant bacterial phylum detected by next-generation sequencing was Proteobacteria (Fig. 2A, left). Commonly represented pathogens included *Staphylococcus*, *Pseudomonas*, and *Klebsiella* (Fig. 2A, right). Commonly represented commensal bacteria included *Corynebacterium*, *Cutibacterium*, and *Bacillus*. Organisms isolated from device swabs plated on microbiological culture are indicated in Table 2. There was considerable overlap between the 16 s raw sequences and the bacterial isolates cultured from the devices, suggesting molecular data were derived from viable bacteria. (Supplementary Fig. 1C).

**Table 2:** Culture growth, identification, and biofilm formation from strains isolated from penile prostheses.

Strain^a^	Number of isolates
Staphylococcus epidermidis	5
Cutibacterium/Proprionibacterium spp	2
Kocuria spp	1
Mycobacterium dioxanotrophicus	1
Streptococcus agalactiae	1
Micrococcus spp	1
Gluconacetobacter	1
Bacillus licheniformis	1

^a^Swabs were taken from all 27 patients. A total of 13 strains were isolated and identified (7 unique patients). 7 of 27 devices had growth on culture (25.9%).

### Microbial abundance by patient factors

**Figure 3:**
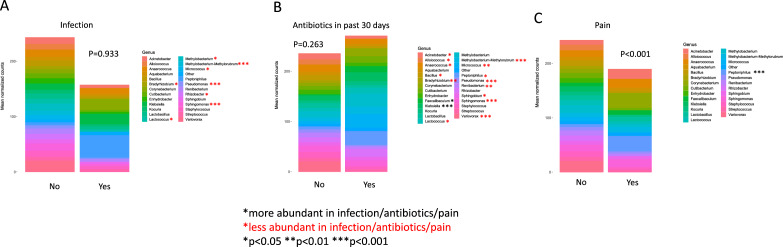
Microbial profile differs by infection and antibiotic status. (**A**) Microbial genera normalized counts stratified by whether or not the analyzed device was associated with infection. (**B**) Microbial normalized counts stratified by antibiotic use within the 30 days prior to device removal. (**C**) Microbial genera normalized counts stratified by whether or not the analyzed device was associated with pain. *p*-values are indicated in respective graphs. Black asterisks indicate genera more abundant in infection/antibiotic use; red asterisks indicate genera less abundant in infection/antibiotic use. *p < 0.05; **p < 0.01; ***p < 0.001.

Total normalized microbial counts were similar by infection status (Fig. 3A). The genera *Faecalibaculum* (log_2_ fold change + 4.10, *p* < 0.001), *Jeotgalicoccus* (+ 3.22, *p* < 0.001), *Nosocomiicoccus* (+ 2.94, *p* < 0.001), *Negativicoccus* (+ 2.50, *p* < 0.001), *Roseomonas* (+ 2.83, *p* < 0.001), *Gemella* (+ 1.94, *p* = 0.012), and *Proprionimicrobium* (+ 1.58, *p* = 0.041) were more abundant in the presence of infection. The genera *Methylobacterium-Methylorubrum* (log_2_ fold change −9.94, *p* < 0.001)*, Sphingomonas* (−6.97, *p* < 0.001)*, Pseudomonas* (−6.52, *p* < 0.001)*, Rhizobacter* (−4.03, *p* = 0.012)*, Micrococcus* (−3.64, *p* = 0.012)*, Methylobacterium* (−3.69, *p* = 0.026)*, Bradyrhizobium* (−3.41, *p* = 0.026)*, Lachnospiracea incertae sedis* (−3.22, *p* = 0.042), and *Lactococcus* (−3.26, *p* = 0.045) were all less abundant in infection.

Total normalized microbial counts were similar between patients who did versus did not use antibiotics within the 30 days prior to device removal (Fig. 3B). *Klebsiella* (log_2_ fold change + 4.4, *p* < 0.001)*, Nosocomiicoccus* (+ 2.44, *p* = 0.002)*, Negativicoccus* (+ 2.04, *p* = 0.010)*, Brevibacterium* (+ 2.10, *p* = 0.020)*, Syntrophaceticus* (+ 1.58, *p* = 0.035)*, Gemella* (+ 1.53, *p* = 0.041)*,* and *Faecalibaculum* (+ 1.87, *p* = 0.048) were more abundant in biofilms from individuals who had antibiotics. Nineteen genera were less abundant in those who had antibiotics within the past 30 days (Fig. 3B).

Penile prostheses with device-associated pain had fewer total normalized microbial counts than those without pain (Fig. 3C, *p* < 0.001). The genera *Thermoanaerobacterium* (log_2_ fold change 4.34, *p* < 0.001), *Murdochiella* (+ 3.55, *p* < 0.001), *Dialister* (+ 4.31, *p* < 0.001), *Peptoniphilus* (+ 4.58, *p* < 0.001), *Prevotella* (+ 3.99, *p* = 0.006), *Propionimicrobium* (+ 2.00, *p* = 0.020), *Candidatus Koribacter* (+ 1.91, *p* = 0.03), and *Tepidimonas* (+ 1.81, *p* = 0.04), were more abundant in the context of pain. No genera were less abundant in the context of pain.

There were fewer total normalized microbial counts from the biofilms from prostheses explanted from current smokers, those with diabetes mellitus, and those with cardiac disease, relative to those without these conditions (Supplementary Fig. 2). Total normalized microbial counts were significantly different among device models (*p* < 0.001).

### Metabolite composition and microbe-metabolite interaction networks

**Figure 4:**
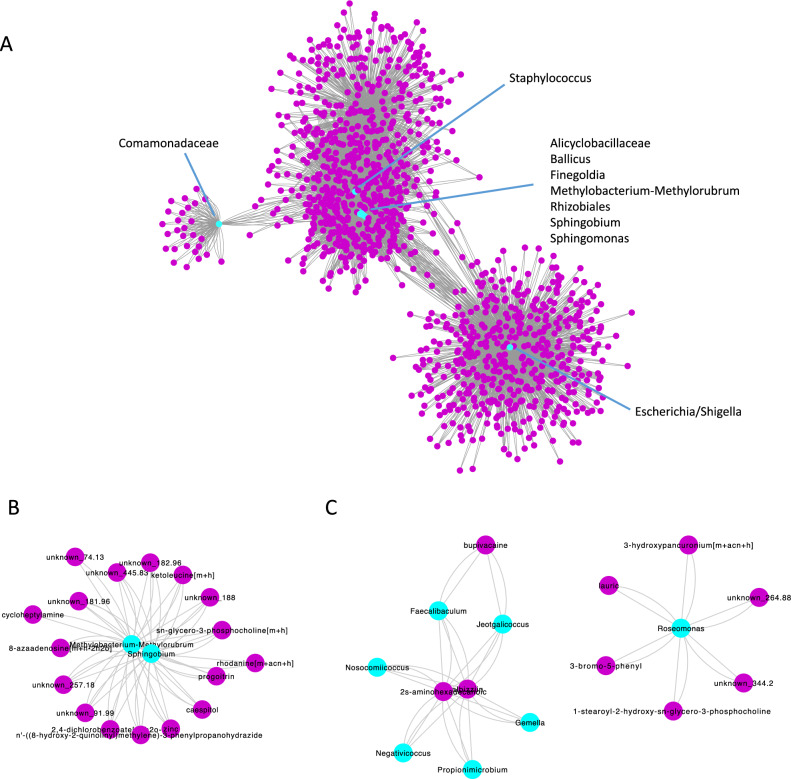
Microbe-metabolite interaction networks. (**A**) Microbe-metabolite interaction network based on relative counts for overall cohort. Microbe-metabolite interaction network enriched in the absence (**B**) and presence (**C**) of device-associated infection. Microbes are indicated in cyan and metabolites are indicated in magenta. Gray lines connect microbes to respective metabolites.

Detected metabolites within device biofilms are shown in Fig. 2B. The most commonly detected metabolites were dimethylamine, 4r-aminopentanoic acid, polypropylene glycol, 3-aminopropane-1,2 diol, and nervonyl carnitine, ammonium acetate, and carbachol. The comprehensive list of detected metabolites is shown in Supplementary Data Set 1. The metabolite diversity of biofilms did not differ as a function of infection status, device-associated pain, antibiotic use in the past 30 days, smoking status, diabetes mellitus, or cardiac disease (Supplementary Fig. 3). The microbe-metabolite network was generated based on microbe and metabolite counts for the entire device population (Fig. 4A) The networks revealed central genera including *Staphylococcus* (2638 metabolites), *Escherichia/Shigella* (1808 metabolites), *Methylobacterium-Methylorubrum* (1380 metabolites), *Bacilli* (1016 metabolites), *Sphingomonas* (974 metabolites), *Finegoldia* (662 metabolites), *Alicyclobacillaceae* (514 metabolites), *Sphingobium* (490 metabolites), *Rhizobiales* (462 metabolites), and *Comamonadaceae* (106 metabolites). Statistically significantly enriched subnetworks were identified for the absence (Fig. 4B) and presence (Fig. 4C) of device-associated infection. Enriched genera in the infection subnetwork included *Faecalibaculum, Jeotgalicoccus*, *Gemella*, *Proprionimicrobium*, *Negativicoccus*, *Nosocomiicoccus*, and *Roseomonas*. Enriched metabolites included 2 s-aminohexadecanoic acid, albizziin, and bupivacaine. *Methylobacterium-Methylorubrum* (32 metabolites) and *Sphingobium* (30 metabolites) were enriched in the subnetwork for absence of infection. There were no statistically significant subnetworks for the presence or absence of pain.

### Biofilm formation of bacterial strains in vitro

**Figure 5:**
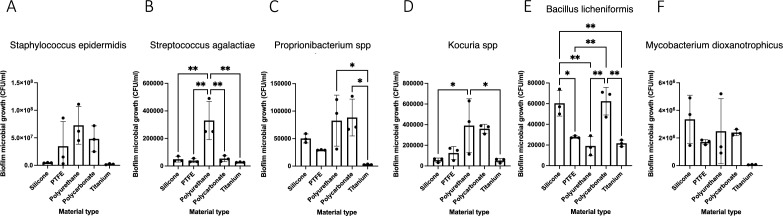
Biofilm deposition varies by strain and material type in vitro. *Staphylococcus epidermidis* (**A**), *Streptococcus agalactiae* (**B**), *Proprionibacterium* (**C**), *Kocuria spp* (**D**), *Bacillus licheniformis* (**E**), or *Mycobacterium dioxanotrophicus* was incubated together in a continuous-flow stir tank bioreactor with a series of coupons of different material types for 72 h. After the incubation period, the coupons were removed and biofilm deposition was assessed through plate count assays. Negative controls, wherein media without strain inoculation was subjected to identical bioreactor conditions were performed, and consistently did not harbor growth on plate count assays. Error bars indicated standard deviation. *p < 0.05, **p < 0.01, ***p < 0.001, ****p < 0.0001.

**Figure 6:**
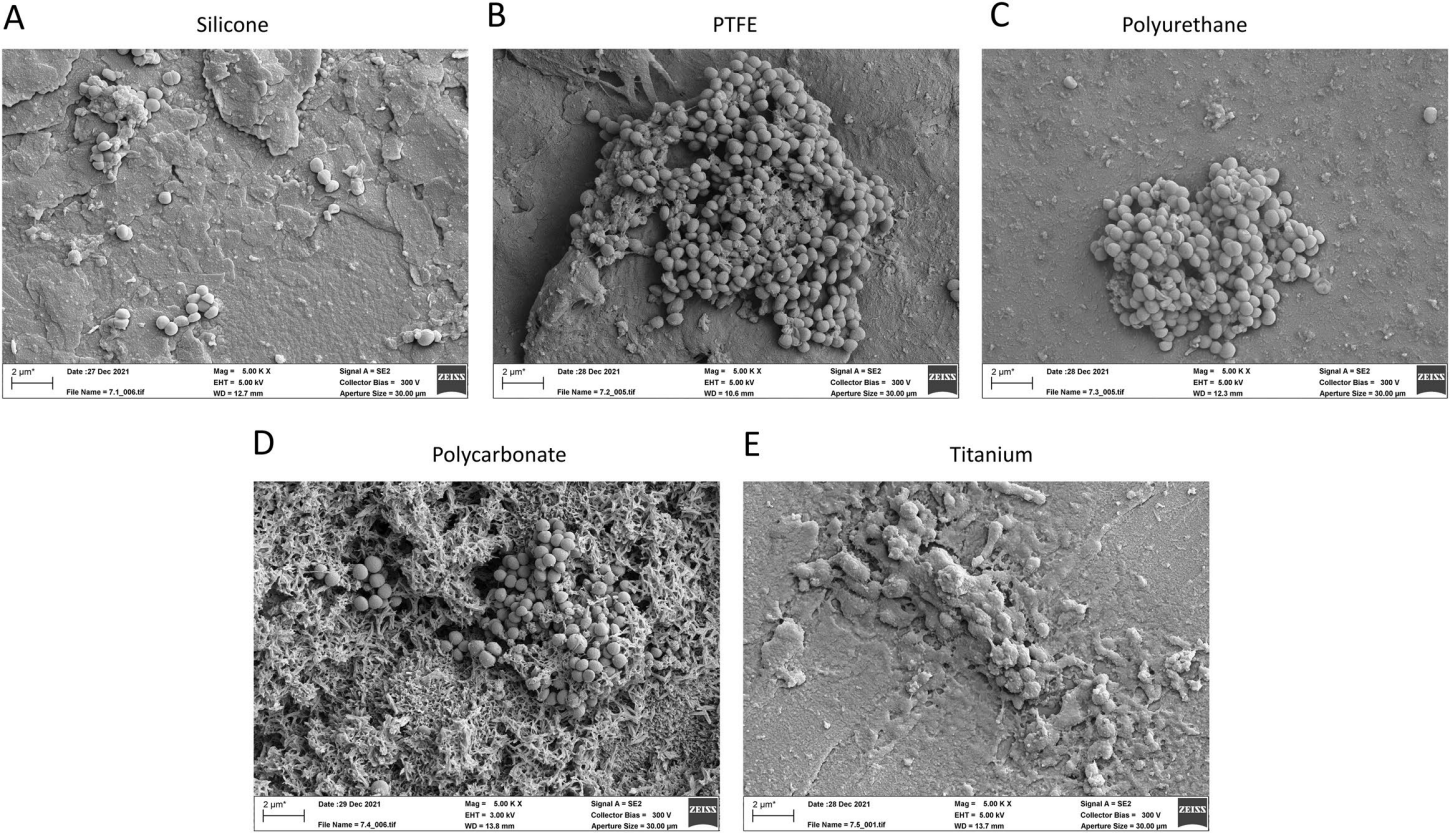
Scanning electron microscopy reveals biofilm formation of *Staphylococcus epidermidis* on multiple material types. Each strain was grown in a continuous-flow stir tank bioreactor for 72 h along with a series of coupons or different material types. Silicone, PTFE, polyurethane, polycarbonate, and titanium are indicated in respective bars. Scale bars are indicated on the respective micrographs.

Based on clinical relevance, culture viability, and importance in the microbe-metabolite interaction networks, a series of microbial strains isolated from prostheses was tested for propensity to form biofilm on various material types in vitro: *Staphylococcus epidermidis*, *Streptococcus agalactiae*, *Proprionibacterium spp*, *Kocuria spp*, *Bacillus licheniformis spp*, and *Mycobacterium dioxanotrophicus*. Tested materials included those commonly-used penile prosthesis and medical device materials: silicone, polytetrafluoroethylene (PTFE), polyurethane, polycarbonate, and titanium. Plate count assays revealed differential biofilm formation both by strain and material type (Fig. 5). *Staphylococcus epidermidis* exhibited greater biofilm formation than other microbial strains (*p* < 0.0001). Biofilm formation was greater on polyurethane than on silicone for both *Streptococcus agalactiae* (*p* < 0.01) and *Kocuria spp* (*p* < 0.05). Conversely, for *Bacillus licheniformis*, biofilm formation was greater on silicone than polyurethane (*p* < 0.01). Scanning electron microscopy showed robust biofilm formation in *Staphylococcus epidermidis* and captured biofilm formation from all other strains, corroborating the plate count assay results (Fig. 6, Supplementary Fig. 4).

### Antibiotic resistance and biofilm gene detection

RT-PCR analysis was performed on 25 of 27 device biofilms. Antibiotic resistance genes were commonly detected in penile prosthesis biofilms. Specifically, *Sul2* (gene encoding for sulfonamide resistance) was present in 48% of samples, *ampC* (encoding for penicillin resistance) in 92% of samples, *tetA* (encoding for tetracycline resistance) in 12% of samples, and an *rpoB* mutation associated with rifampin resistance in 36% of samples. The *fimH* adhesin gene, implicated in biofilm formation, was detected in 48% of samples. The detection of antibiotic resistance genes or *fimH* gene did not differ by infection status, antibiotic use in the past 30 days, pain status, or device type. The *rpoB* mutation was detected in biofilms from 35% of IPPs with minocycline/rifampin coating, 25% of IPPs with surgeon-defined antibiotic dip, and 50% of malleable devices (*p* = 0.76). *tetA* was detected in biofilms from 5.9% of IPPs with rifampin/minocycline coating, 25% of IPPs with surgeon-defined antibiotic irrigation, and 12% of malleable prostheses (*p* = 0.39).

## Discussion

We characterized the biofilm quantity and composition of penile prosthesis devices. We demonstrated that biofilm formation is present across the great majority of explanted devices, even in the absence of infection. Together, the data support a paradigm shift in the understanding of prosthesis associated infection: sterile penile prostheses are the exception, rather than the rule, and whether attempts to eradicate this ubiquitous biofilm are beneficial and/or attainable requires investigation. Inhibiting the transition to dysbiosis (imbalance among microbiota in a niche) rather than complete removal of both helpful and harmful bacteria alike may be the optimal strategy to avoid infection. There is mounting evidence that, even in the asymptomatic state, biofilms may interact with the host, potentially contributing to pathological and immunological sequelae including systemic autoimmune disease and neurodegenerative disease^14^. Future studies are needed to understand the relationship between medical device biofilm and such non-infectious pathology, and whether biofilm modulation could affect outcomes.

Our data show biofilm diversity increases as a function of device indwelling time, continuing to evolve even decades after implantation and in the absence of evolution to clinical infection. This increased diversity may in fact provide a protective effect and explain the finding of reduced infectious risk with increased indwelling time^15^. We identified specific microbial genera including *Faecalibaculum, Klebsiella,* and *Thermoanaerobacterium* that were differentially abundant in the context of device-associated infection, recent antibiotic use, and device-associated pain, respectively. Additionally, we demonstrated that biofilms from metabolically and clinically important microbial strains isolated from explanted penile prostheses could consistently be reconstituted in vitro.

Surprisingly, there was also a lower bacterial abundance in individuals with diabetes mellitus. This suggests the mechanism underlying this leading risk factor for infection may in fact be a dysregulated network of taxa that lack ‘protective’ symbiotic bacteria capable of inhibiting infection, rather than bacterial overgrowth of harmful pathogens.

The commonly-used rifampin and minocycline commercial coating for penile prostheses has been associated with device-associated infection reduction in historical case series^16^, but we detected relevant antibiotic resistance genes in a large proportion of analyzed devices. Of note, an *rpoB* mutation consistent with rifampin resistance was detected in 36% of devices including those without clinical infection. This finding underscores the importance of resistance and raises the possibility that extended exposure in situ may predispose bacteria to developing resistance. This finding may have important consequences in the setting of device replacement, which is known to carry an increased risk of infection compared to index cases. In contrast, literature suggests similar infection rates between the precoated and surgeon-coated devices, again underscoring the complex nature of microbial symbiosis and dysbiosis. The finding also highlights that while antibiotics may play a role in the prevention of the dysbiotic biofilm state, novel alternatives to antibiotics may present an option going forward and warrant further investigation.

*Staphyloccocus* and *Escherichia/Shigella* were the most metabolically-central genera in penile prosthesis devices. This is consistent with the finding that viable *Staphylococcus epidermidis* is present in up to 40% of uninfected penile prosthesis devices and that *Escherichia coli* and *Staphylococcus* are the most common organisms isolated in penile prosthesis infection^17,18^. Interestingly, *Staphylococcus* was not overly represented in the presence of pain or infection, nor was it enriched in the infection or non-infection subnetworks. *Escherichia* also was not differentially abundant or enriched in the infection or non-infection subnetwork. These findings together support the hypothesis that a pattern of microbes and metabolites, rather than overgrowth of a single strain, is responsible for device-associated infection or pain. It also raises the intriguing possibility that while these microbes are identified via traditional culture and held responsible for clinical infection, this is clearly not always the case.

In infected devices, the enriched microbe-metabolite subnetwork identified 2 s-aminohexadecanoic acid, an alpha-amino fatty acid. This metabolite has shown to be upregulated following bacterial treatment with hydroxybenzoic acid^19^. In addition, bupivacaine, an anesthetic commonly used for nerve blocks, was a centrally enriched metabolite in the context of infection. The source of bupivacaine in biofilms in this study is not clear, as its use in revision surgeries is not routine in the authors’ practice, but its enrichment in the context of infection could be related to its use as local anesthetic, perhaps during the initial prosthetic implantation. It is plausible that percutaneous introduction of local anesthetic provides an additional nidus for infection, or that the molecule itself dysregulates a microbe-metabolite interaction network, facilitating infection. The study is limited in that it was not designed to detect fungi, which may also play a role in penile prosthesis biofilm formation^18^. An additional limitation is the relatively low sample size of infected devices, which may have been associated with the lack of detectable differences by infection status. Further, in many cases there is overlap between the signs and symptoms of infection and those of pain. Thus, it is possible that some devices that were categorized in the present study as “explanted due to pain” may also have had a subclinical infectious component. Another limitation was that the study only resolved microbiota to the genus level. There may be phenotypic differences within genera, and in some cases both pathogenic and commensal species exist in a single genus. Despite use of the latest 16S rRNA Silva and NCBI databases, as well as assigning taxa to the strain level (based on amplicon sequence variants), species were only able to be assigned to 17.8% of ASVs. This is likely because the penile prosthesis is a novel niche to study the microbiome and likely harbors many novel species. The NCBI and Silva databases are largely based on gut bacteria and other common environmental bacteria and are thus limited in that capacity. We note that this limitation is not unique to the present study, but rather is currently the case for the microbiome field in general. Evidence in related subjects such as orthopedic trauma and urinary tract infections, suggests that there is currently insufficient evidence for NGS as a substitute or complement to conventional culture, and additional studies are necessary to determine its clinical utility^20,21^. Although there was considerable overlap between the NGS and cuture results in device biofilms in the present study, the clinical utility of NGS for the diagnosis of device-associated infection or to inform treatments for this condition requires further investigation. Despite these limitations, our study has several strengths. First, it used a multi-pronged approach to detected microbiota both with high-throughput culture-independent and culture-based assays. Further, the study provides a robust characterization of isolated microbial strains, both in identification and ability to reconstitute biofilm formation in vitro in an environment mimicking human tissue with an indwelling medical device. Biofilm formation was measured using plate count assays and corroborated with scanning electron microscopy. The findings and approaches together have major implications for the future development of infection prevention strategies.

## Conclusions

Penile prosthesis device biofilms ubiquitously harbor microbiota in the presence or absence of infection, and antibiotic resistance genes were commonly detected. *Staphylococcus* and *Escherichia* are central in microbe-metabolite interaction networks but exhibited similar abundance irrespective of infection or pain status. Other specific microbial genera and metabolites were differentially-abundant in the context of pain and infection, suggesting a microbe-metabolite pattern, rather than overgrowth of a specific strain, is responsible for the transition from the asymptomatic state to the infectious or painful state. Metabolically and clinically important microbial strains consistently reconstituted biofilm in vitro, with differential formation by strain and material type*.* Our work provides a new framework for understanding penile prosthesis infection and supports the notion that downregulation of symbiotic bacteria, rather than overproduction of dysbiotic bacteria, may be the key inciting step in penile prosthesis infection.

## Supplementary Information

Supplementary Information 1.

Supplementary Information 2.

Supplementary Figures.

## Data Availability

The datasets used and/or analysed during the current study available from the corresponding author on reasonable request. All figures and tables herein are original and not reproduced from other sources.
