# Supplementary material for: Microbe-metabolite interaction networks, antibiotic resistance, and in vitro reconstitution of the penile prosthesis biofilm support a paradigm shift from infection to colonization
Source: Sci Rep. 2023 Jul 17;13:11522. doi: 10.1038/s41598-023-38750-1 (PMC10352235; doi:10.1038/s41598-023-38750-1)
Supplement: Supplementary file 1 — Supplementary Information 1. [file 41598_2023_38750_MOESM1_ESM.docx]

**Supplemental Methods**

*Sample processing*

The swabbed specimens were maintained at 4°C and transferred to -80°C within 4 hours of collection. Samples and negative and positive controls were subjected to 16S next-generation sequencing, metabolomics, and culture-based approaches to determine microbial composition and diversity, and their association with clinical factors.

DNA and metabolites were extracted from the swabs using standard, validated approaches previously described.^1-3^ Briefly, for DNA extraction, bacteria were dislodged from the first swab in a sterile PBS solution with agitation. The lysis protocol included bead-beating, piston-driven lysis, and proteinase K treatment. DNA was attached to magnetic beads in salt solution, and was removed from solution and subsequently eluted in buffer.

For metabolomics, the second swab was similarly agitated in sterile PBS. Each sample was diluted in acetonitrile solution containing 4-nitrobenzoic acid (Acros Organics, Fair Lawn, NJ, USA), and debrisoquine (Santa Cruz Biotechnology, Dallas, TX, USA) internal standards.^1,4^ Centrifugation was performed to precipitate proteins and the supernatant was recovered and stored at −80 °C prior to analysis.^5^

For culture, the third swab was streaked on a series of agar plates of four compositions: Blood agar, CNA agar, Chocolate agar, and MacConkey agar. The media reflected the conditions used by the clinical microbiology lab at our institution. Plates were incubated at temperature of 37°C for 5 days. Colonies were next transferred to subsequent plates for isolation, and isolated colonies were then transferred to liquid BHI culture and grown to logarithmic or stationary phase. DNA extraction from the culture was performed similarly to that of the first swab. Culture glycerol stocks were prepared and stored at -80 °C until analysis.

*Sequencing*

DNA samples were submitted to the Microbial Sequencing and Analytics Core at our institution for high throughput sequencing of the 16S rRNA gene on an Illumina iSeq. DNA was amplified with PCR with 515F and 806R primers targeting V4 hypervariable region of the 16S gene as previously described.^3^ DNA concentration was quantified prior to and after PCR amplification and normalized before library prep with the Illumina Nextera XT library kit. Sequence runs were conducted to generate 150bp, paired-end sequences. Duplicate samples of a DNA standard (Zymobiomics, USA) were included as positive controls, as well as the DNA reagents that were subjected to the entire workflow, sterile water, and a PCR negative as negative controls.

DNA extracted from culture bacterial isolates from the third swab was amplified with PCR using 27F and 1492R primers and Sanger sequencing by the genomics core at our institution. Sequences were taxonomically assigned via BLAST.

*Antibiotic resistance and adhesin gene detection*

Antibiotic resistance genes *ampC* (associated with penicillin resistance)*, sul2* (associated with sulfonamide resistance), *tetA* (associated with tetracycline resistance), and a mutation in the *rpoB* gene associated with rifampin resistance*,* and the pilus adhesin gene *fimH* (associated with biofilm formation), were detected using reverse transcriptase PCR (rtPCR) as described^6^, using a universal bacterial primer as a standard. Primers for detection were previously reported and used as described.^7-11^

*Metabolomics*

Metabolite fractions, prepared as above, were processed by the Metabolomics Core at our institution. Metabolomics was performed as previously described.^1,5^ Briefly, liquid chromatography/tandem mass spectroscopy (LC-MS-MS) was used. External standards were added to the samples. Samples were injected onto the Vanquish UHPLC system coupled to the Q Exactive HF hybrid quadrupole-orbitrap mass spectrometer (Thermo Scientific, Waltham, MA). Operation was in positive and negative electrospray ionization modes in the mass range 50–750 Da. Raw data deconvolution was performed with XCMS software.^12^ Detected ions were normalized to creatinine. Metabolyzer software was used for further analysis.^13^ Concentration quantification was performed through comparison to the two added internal standards. Metabolites were identified by ionization, molecular mass, and retention time (m/z). Putative identity assignment was performed through comparison to metabolites present in KEGG, HMDB, LIPIDMAPS, and BioCyc databases.^14-17^

*Bioinformatics*

The 16s sequencing data as obtained above were processed in R statistical packages unless otherwise noted. Bioinformatic analysis of 16s data was performed as previously described.^3^ Dada2 was used for quality control, bimera removal, and amplicon sequence variants (ASV) assignment.^18^ A combined database of Silva 138 SSURef^19^ and NCBI 16S rRNA^20^ was used for ASV assignment. MSA was used for ASV alignment,^21^ and ASVs were arranged into a maximum likelihood phylogeny in phangorn.^22^ The resulting phylogenetic tree was then combined with the ASV table. This was merged with sample data for loading into PhyloSeq and taxa assigned to chloroplasts, mitochondria, or eukaryotes were removed.^23^ Sequencing depth threshold necessary to adequately capture microbial diversity was calculated in Vegan with a rarefaction analysis.^24^ Samples below the empirically determined depth threshold were removed from further analysis. Contamination removal was performed using Decontam^25^ with negative controls as the source.

The resulting count table of decontaminated and remaining high-quality reads was normalized using DESeq2^26^, which executes a negative binomial Wald test to minimize differences based on sequencing depth, while maintaining rare taxa. From the normalized table, α-diversity was next calculated using the phylogenetic diversity metric in Phyloseq. This quantified the number of unique phylogenetic groups in a sample. Beta-diversity was calculated as a weighted UniFrac distance.^27^ The weighted UniFrac metric quantifies differences in microbial communities based on presence/absence of phylogenetic groups along with their relative abundance. Beta-diversity statistical analyses were conducted with PERMANOVA, after 999 permutations. Alpha diversity was analyzed using paired t*-*test with Holm’s correction, where applicable. The DESeq2 algorithm was used to determine which ASV’s differentiated samples.^26^ To validate that the high throughput sequencing data originated from viable bacteria in the biofilms, sequences from isolated bacteria were mapped against the high throughput sequencing data.

Normalized metabolite concentrations from positive and negative electron spray ionization modes were batch-corrected using the BER package.^28^ This uses a linear regression model to locate and scale batch effects, while maintaining treatment effects when present. The algorithm has been validated for untargeted metabolomic data derived from urinary samples.^5^ Metabolome composition dissimilarity based on metadata categories was quantified using a weighted Binomial dissimilarity matrix, which is the optimal assessment for dissimilarity in metabolomic data.^29^ Statistical analysis of dissimilarity matrices was conducted using a PERMANOVA with 999 permutations in the VEGAN package.^24^ A permuted Welch’s t-test with Benjamini-Hochberg correction was conducted to determine specific metabolites that differentiated metadata categories.

Multi-omic integration of 16S rRNA and untargeted metabolomic data was performed through the calculation all pairwise microbe-metabolite Pearson correlations. Correlations >0.4 with false-discovery corrected p-values <0.05 were used in downstream analyses. Network visualization was provided in Cytoscape.^30^

*Continuous-flow stir tank bioreactor biofilm assays and scanning electron microscopy*

A continuous stir-tank CDC bioreactor was used to reconstitute and quantify *in vitro* biofilm formation from device-isolated bacteria on a series of medical device materials as previously described.^31^ Materials included silicone, PTFE, polyurethane, polycarbonate, and titanium. The bioreactor is a culture tank with controlled conditions including temperature, turbulence, and flow rate, designed to mimic those of human tissue with an indwelling medical device present. Briefly, the culture tank was filled with BHI media and inoculated with a microbial strain grown to logarithmic or stationary phase. In the case of negative controls, BHI media was added to the culture tank under identical conditions, but without inoculation with a microbial strain. Temperature was 37 °C and culture flow rate was 1 ml/min for strains and negative controls. Each strain and control was incubated in the reactor with the above material coupons for 72 hours. Each device material type for a given microbial strain was included in triplicate. Following the 72 hour incubation period, coupons were retrieved and rinsed in PBS. Next, biofilm adherent to coupons were resuspended using a combination of scraping of all surfaces and agitation. Resultant solutions were then plated in a series of tenfold dilutions on BHI agar plates overnight. Plate colony counts were then conducted using standard approaches. Differences among materials and strains were analyzed with ANOVA and Bonferroni t-tests. Plate count assay figure images were generated using GraphPad Prism 9 (GraphPad Software, www.graphpad.com).

A subset of coupons was subjected to scanning electron microscopic analysis. Coupons were rinsed in PBS solution and fixed in 4% formalin solution. They were then serially dehydrated in ethanol and stained with osmium tetroxide. They were submitted to the Imaging Core at our institution for processing. Fixed, dehydrated samples were mounted and gold sputter coated, followed by imaging on a Zeiss Sigma VP scanning electron microscope.^31^

References

1. Zampini A, Nguyen AH, Rose E, Monga M, Miller AW. Defining dysbiosis in patients with urolithiasis. *Scientific reports*. 2019;9(1):1-13.

2. Kachroo N, Lange D, Penniston KL, et al. Standardization of microbiome studies for urolithiasis: an international consensus agreement. *Nature Reviews Urology*. 2021;18(5):303-311.

3. Werneburg GT, Adler A, Zhang A, et al. Transperineal prostate biopsy is associated with lower tissue core pathogen burden relative to transrectal biopsy: mechanistic underpinnings for lower infection risk in the transperineal approach. *Urology*. 2022;

4. Goudarzi M, Weber W, Mak TD, et al. Development of urinary biomarkers for internal exposure by cesium-137 using a metabolomics approach in mice. *Radiation research*. 2014;181(1):54-64.

5. Agudelo J, Fedrigon D, Faris A, Wilkins L, Monga M, Miller AW. Delineating the role of the urinary metabolome in the lithogenesis of calcium-based kidney stones. *Urology*. 2022;

6. Livak KJ, Schmittgen TD. Analysis of relative gene expression data using real-time quantitative PCR and the 2− ΔΔCT method. *methods*. 2001;25(4):402-408.

7. Stedtfeld RD, Guo X, Stedtfeld TM, et al. Primer set 2.0 for highly parallel qPCR array targeting antibiotic resistance genes and mobile genetic elements. *FEMS Microbiology Ecology*. 2018;94(9):fiy130.

8. Suzuki MT, Taylor LT, DeLong EF. Quantitative analysis of small-subunit rRNA genes in mixed microbial populations via 5′-nuclease assays. *Applied and environmental microbiology*. 2000;66(11):4605-4614.

9. Pusz P, Bok E, Mazurek J, Stosik M, Baldy-Chudzik K. Type 1 fimbriae in commensal Escherichia coli derived from healthy humans. *Acta Biochimica Polonica*. 2014;61(2)

10. Nõlvak H, Truu M, Tiirik K, et al. Dynamics of antibiotic resistance genes and their relationships with system treatment efficiency in a horizontal subsurface flow constructed wetland. *Science of the total environment*. 2013;461:636-644.

11. Torres MJ, Criado A, Palomares JC, Aznar J. Use of real-time PCR and fluorimetry for rapid detection of rifampin and isoniazid resistance-associated mutations in Mycobacterium tuberculosis. *Journal of clinical microbiology*. 2000;38(9):3194-3199.

12. Tautenhahn R, Patti GJ, Rinehart D, Siuzdak G. XCMS Online: a web-based platform to process untargeted metabolomic data. *Analytical chemistry*. 2012;84(11):5035-5039.

13. Mak TD, Laiakis EC, Goudarzi M, Fornace Jr AJ. MetaboLyzer: a novel statistical workflow for analyzing postprocessed LC–MS metabolomics data. *Analytical chemistry*. 2014;86(1):506-513.

14. Kanehisa M, Sato Y, Furumichi M, Morishima K, Tanabe M. New approach for understanding genome variations in KEGG. *Nucleic acids research*. 2019;47(D1):D590-D595.

15. Wishart DS, Feunang YD, Marcu A, et al. HMDB 4.0: the human metabolome database for 2018. *Nucleic acids research*. 2018;46(D1):D608-D617.

16. Fahy E, Alvarez-Jarreta J, Brasher CJ, et al. LipidFinder on LIPID MAPS: peak filtering, MS searching and statistical analysis for lipidomics. *Bioinformatics*. 2019;35(4):685-687.

17. Caspi R, Altman T, Billington R, et al. The MetaCyc database of metabolic pathways and enzymes and the BioCyc collection of Pathway/Genome Databases. *Nucleic acids research*. 2014;42(D1):D459-D471.

18. Callahan BJ, McMurdie PJ, Rosen MJ, Han AW, Johnson AJA, Holmes SP. DADA2: High-resolution sample inference from Illumina amplicon data. *Nature methods*. 2016;13(7):581-583.

19. Quast C, Pruesse E, Yilmaz P, et al. The SILVA ribosomal RNA gene database project: improved data processing and web-based tools. *Nucleic acids research*. 2012;41(D1):D590-D596.

20. Hrabek-Smith JM, Carroll KK. A comparative study of serum lipoproteins in rabbits fed a natural ingredient diet or low-fat, cholesterol-free, semipurified diets containing casein or isolated soy protein. *Biochemistry and Cell Biology*. 1987;65(7):610-616.

21. Bodenhofer U, Bonatesta E, Horejš-Kainrath C, Hochreiter S. msa: an R package for multiple sequence alignment. *Bioinformatics*. 2015;31(24):3997-3999.

22. Schliep KP. phangorn: phylogenetic analysis in R. *Bioinformatics*. 2011;27(4):592-593.

23. McMURDIE PJ, Holmes S. Phyloseq: a bioconductor package for handling and analysis of high-throughput phylogenetic sequence data. *Biocomputing 2012*. World Scientific; 2012:235-246.

24. Oksanen J, Blanchet FG, Friendly M, et al. Vegan: community ecology package (version 2.5-6). *The Comprehensive R Archive Network*. 2019;

25. Davis NM, Proctor DM, Holmes SP, Relman DA, Callahan BJ. Simple statistical identification and removal of contaminant sequences in marker-gene and metagenomics data. *Microbiome*. 2018;6(1):1-14.

26. Love MI, Huber W, Anders S. Moderated estimation of fold change and dispersion for RNA-seq data with DESeq2. *Genome biology*. 2014;15(12):1-21.

27. Wong RG, Wu JR, Gloor GB. Expanding the UniFrac toolbox. *PloS one*. 2016;11(9):e0161196.

28. Saama PM, Patel OV, Bettegowda A, Ireland JJ, Smith GW. Novel algorithm for transcriptome analysis. *Physiological genomics*. 2006;28(1):62-66.

29. Qi Z, Voit EO. Strategies for comparing metabolic profiles: implications for the inference of biochemical mechanisms from metabolomics data. *IEEE/ACM transactions on computational biology and bioinformatics*. 2016;14(6):1434-1445.

30. Smoot ME, Ono K, Ruscheinski J, Wang P-L, Ideker T. Cytoscape 2.8: new features for data integration and network visualization. *Bioinformatics*. 2011;27(3):431-432.

31. Hobbs T, Schultz LN, Lauchnor EG, Gerlach R, Lange D. Evaluation of biofilm induced urinary infection stone formation in a novel laboratory model system. *The Journal of urology*. 2018;199(1):178-185.
