# Supplementary material for: Microbe-metabolite interaction networks, antibiotic resistance, and in vitro reconstitution of the penile prosthesis biofilm support a paradigm shift from infection to colonization
Source: Sci Rep. 2023 Jul 17;13:11522. doi: 10.1038/s41598-023-38750-1 (PMC10352235; doi:10.1038/s41598-023-38750-1)
Supplement: Supplementary file 3 — Supplementary Figures. [file 41598_2023_38750_MOESM3_ESM.docx]

**Supplementary Figure 1: Bioinformatic controls.** Alpha (A) and beta (B) diversity by sample type. P-values are indicated in each respective panel. Positive controls include commercial DNA standards and a laboratory cultured urinary bacteria. Negative controls included DNA extraction reagents, and a PCR negative control. (C) Mapping of 16s sequencing to sequencing of cultured isolates reveals good overlap.

**Supplementary Figure 2: Microbial profile differs by patient factors.** Microbial normalized counts stratified by patient factors. Patient factors are indicated on the respective X-axis. P-values are indicated in respective panels.

**Supplementary Figure 3: Metabolite diversity by patient factors.** Metabolite diversity stratified by patient factors is shown by principal components analysis. Factors are indicated in the respective panels. P-values are indicated in respective panels.

**Supplementary Figure 4: Scanning electron microscopy reveals biofilm formation on multiple strains and material types.** Each strain was grown in a continuous-flow stir tank bioreactor for 72 hours along with a series of coupons or different material types. Each column represents a coupon material type and each row is a specific isolate. Scale bars are indicated on the respective micrographs. Negative controls, wherein media without microbial strain was incubated in the bioreactor, are indicated by material type in the right-most column.
